# BmK86-P1, a New Degradation Peptide with Desirable Thermostability and Kv1.2 Channel-Specific Activity from Traditional Chinese Scorpion Medicinal Material

**DOI:** 10.3390/toxins13090610

**Published:** 2021-08-30

**Authors:** Chenhu Qin, Xuhua Yang, Zheng Zuo, Liuting Yang, Fan Yang, Zhijian Cao, Zongyun Chen, Yingliang Wu

**Affiliations:** 1 College of Life Sciences, Wuhan University, Wuhan 430072, China; 2016202040025@whu.edu.cn (C.Q.); yangxh@whu.edu.cn (X.Y.); 2014301060068@whu.edu.cn (Z.Z.); 2018202040126@whu.edu.cn (L.Y.); y_fan@whu.edu.cn (F.Y.); zjcao@whu.edu.cn (Z.C.); chenzy2005@126.com (Z.C.); 2 Department of Biochemistry and Molecular Biology, College of Basic Medicine, Hubei University of Medicine, Shiyan 442000, China; 3 Center for BioDrug Research, Wuhan University, Wuhan 430072, China

**Keywords:** thermally processed scorpions, BmK86-P1, electrophysiology experiments, Kv1.2 channel inhibitor, bioactive peptide

## Abstract

Thermally processed *Buthus martensii* Karsch scorpions are a traditional Chinese medical material for treating various diseases. However, their pharmacological foundation remains unclear. Here, a new degraded peptide of scorpion toxin was identified in Chinese scorpion medicinal material by proteomics. It was named BmK86-P1 and has six conserved cysteine residues. Homology modeling and circular dichroism spectra experiments revealed that BmK86-P1 not only contained representative disulfide bond-stabilized α-helical and β-sheet motifs but also showed remarkable stability at test temperatures from 20–95 °C. Electrophysiology experiments indicated that BmK86-P1 was a highly potent and selective inhibitor of the hKv1.2 channel with IC_50_ values of 28.5 ± 6.3 nM. Structural and functional dissection revealed that two residues of BmK86-P1 (i.e., Lys^19^ and Ile^21^) were the key residues that interacted with the hKv1.2 channel. In addition, channel chimeras and mutagenesis experiments revealed that three amino acids (i.e., Gln^357^, Val^381^ and Thr^383^) of the hKv1.2 channel were responsible for BmK86-P1 selectivity. This research uncovered a new bioactive peptide from traditional Chinese scorpion medicinal material that has desirable thermostability and Kv1.2 channel-specific activity, which strongly suggests that thermally processed scorpions are novel peptide resources for new drug discovery for the Kv1.2 channel-related ataxia and epilepsy diseases.

## 1. Introduction

Processed *Buthus martensii* Karsch scorpions are traditional Chinese medical material for treating many diseases, such as chronic pain, rheumatoid arthritis, and apoplexy [1]. Traditionally, the processed scorpions were regarded as effective for disease treatment based on the Chinese saying “combat poison with poison”. Today, mass spectrometry-based proteomics has revealed that there is a large number of wild-type peptides and novel degraded peptides that have the potential to act on potassium channels in processed scorpion medicinal material [2], and some of these classical neurotoxins and degraded peptides have been shown to be potent potassium channel inhibitors, such as BmKKx2, BmKTX, BmKcug2 and BmKcug2-P1 [2,3]. In view of the close relationship between potassium channels and diseases [4,5,6,7], these channel-blocking peptides are increasingly considered to be new drug leads for Kv channelopathies [6,8]. Clearly, it is essential to characterize the potential potassium channel peptide inhibitors in processed scorpion medicinal materials extensively.

In this study, we identified a novel potassium channel-inhibiting BmK86 peptide analog, BmK86-P1, from the scorpion medicinal material by proteomics. The gene encoding toxin BmK86 was found in a cDNA library of *Mesobuthus martensii* Karsch. Bioinformatics analyses showed that it encodes a signal peptide of 22 amino acid residues and a mature toxin of 35 residues with three disulfide bridges. Pharmacological experiments revealed that the recombinant BmK86 peptide could inhibit the current of Kv1.3 channel with IC_50_ values of 150 ± 57 nM [9]. Here, pharmacological experiments showed that BmK86-P1 selectively exhibited a high affinity for the human Kv1.2 channel with IC_50_ values of 28.5 ± 6.3 nM. The site-directed mutagenesis and channel chimera experiments illustrated the key amino acids of BmK86-P1 interacting with the human Kv1.2 channel and the channel critical residues responsible for the BmK86-P1 toxin selectivity. In conclusion, this study not only found the novel Kv1.2-selective peptide BmK86-P1 from the scorpion medicinal material but also clarified the mechanism by which BmK86-P1 interacted with the human Kv1.2 channel, which was helpful for unveiling the medicinal active ingredients of the traditional Chinese scorpion medicinal material.

## 2. Results

### 2.1. Identification of a New Peptide BmK86-P1 from Scorpion Medicinal Material

**Figure 1:**
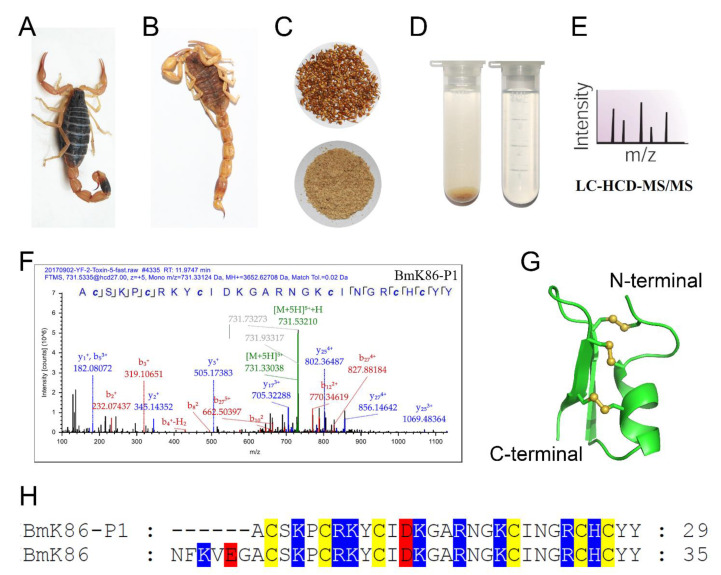
Discovery of a new degraded peptide, BmK86-P1, with six conserved cysteine residues from the processed traditional Chinese medicine *Buthus martensii* Karsch. (**A**) *Buthus martensii* Karsch. (**B**) Thermally processed scorpion. (**C**) The telsons of thermally processed scorpions were ground into powder. (**D**) The powder of the telsons was mixed with ddH_2_O at room temperature for over 24 h, and then the supernatant was filtered by a 0.22 μm filter to obtain a liquid extract of the telsons. (**E**) The telson extract was subjected to MS/MS spectra analysis. (**F**) Identification of BmK86-P1 by MS/MS spectra analysis. The amino acid sequence of BmK86-P1 was confirmed through analyzing the b-ions and y-ions and their derivatives, which were shown in red and blue, respectively. The amino acid sequence of BmK86-P1 was listed together with the sequence coverage. (**G**) The 3D structure of BmK86-P1. The structure of BmK86-P1 was modelled through the SWISS-MODEL server according to the Mesomartoxin template (PDB code: 2RTZ). (**H**) Sequence alignment of BmK86-P1 and BmK86. The background colors of cysteines, acidic residues, and basic residues were colored by yellow, red and blue, respectively.

The traditional Chinese scorpion medicine materials have been used for over one thousand years. There are many peptides with or without conserved cysteine-stabilized disulfide bonds in processed *Buthus martensii* Karsch scorpions [2]. Here, we further identified a novel peptide, BmK86-P1, with six conserved cysteines in processed scorpion medicinal material through proteomic analysis (Figure 1A–F,H). The detected b-ions and y-ions of BmK86-P1 peptide detected in the spectra revealed the presence of BmK86-P1 peptide (Figure 1F). The 3D structure of BmK86-P1 was predicted by SWISS-MODEL (Figure 1G), which indicated that BmK86-P1 could form a stable structure with a cysteine-stabilized α-helical and β-sheet (CSαβ) fold.

**Figure 2:**
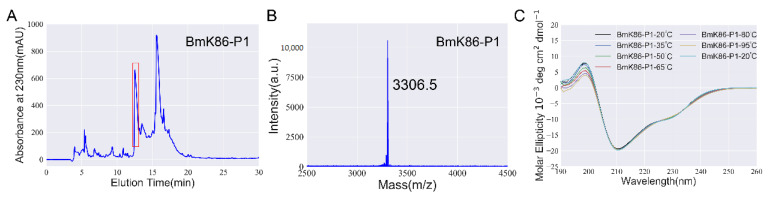
Production and thermal stability analysis of BmK86-P1 peptide. (**A**) Peak profile of BmK86-P1 as purified by high-performance liquid chromatography. The fraction containing BmK86-P1 is indicated by the red box. (**B**) Mass spectrometry analysis of the BmK86-P1. (**C**) Circular dichroism spectra of BmK86-P1 at 20 °C, 35 °C, 50 °C, 65 °C, 80 °C and 95 °C. The CD spectra was done for peptide before heating, after heating and after cooling down at 20 °C.

To investigate the pharmacological properties of BmK86-P1, recombinant BmK86-P1 was successfully produced by a prokaryotic expression system. BmK86-P1 was collected manually by high performance liquid chromatography at 13–14 min (Figure 2A). Then, the peptide was further analyzed by mass spectrometry. The measured molecular weight of BmK86-P1 was 3306.5 Da (Figure 2B), which was consistent with its calculated value of 3305.9 Da. This result indicated that the recombinant BmK86-P1 peptide was successfully obtained for the following experiments.

The peptide BmK86-P1 was found in the thermally processed scorpions, which might imply that BmK86-P1 was a thermally stable peptide. In order to characterize the thermal stability of BmK86-P1, we analyzed the secondary structure of BmK86-P1 peptide in aqueous solutions at different temperatures (20 °C, 35 °C, 50 °C, 65 °C, 80 °C and 95 °C) by CD spectroscopy. The results indicated that the secondary structures of the BmK86-P1 peptide changed only slightly at different temperatures, and the secondary structure was exactly the same as that before heating when the temperature was returned to 20 °C (Figure 2C). This result suggested that BmK86-P1 was thermally stable, which explained why BmK86-P1 remained in the thermally processed scorpions.

### 2.2. Electrophysiological Activities of BmK86-P1

**Figure 3:**
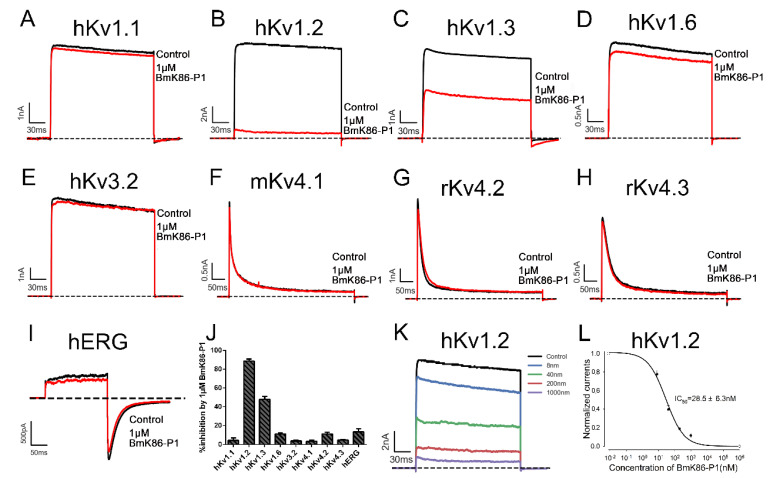
Inhibitory effects of BmK86-P1 on potassium channels. (**A**–**I**) Blocking effects of 1 μM BmK86-P1 on hKv1.1, hKv1.2, hKv1.3, hKv1.6, hKv3.2, mKv4.1, rKv4.2, rKv4.3 and hERG channels. (**J**) The inhibitory rates of 1 μM BmK86-P1 on hKv1.1, hKv1.2, hKv1.3, hKv1.6, hKv3.2, mKv4.1, rKv4.2, rKv4.3 and hERG channels. The percentages were 4.4 ± 2.6% for hKv1.1, 88.6 ± 2.2 for hKv1.2, 52.1 ± 3.1% for hKv1.3, 11.0 ± 1.2% for hKv1.6, 3.8 ± 0.7% for hKv3.2, 3.4 ± 1.1% for mKv4.1, 11.0 ± 1.8% for rKv4.2, 4.9 ± 0.3% for rKv4.3 and 13.3 ± 3.3 for hERG. (**K**) Blocking effects of different concentrations of BmK86-P1 on the hKv1.2 channel. (**L**) Average normalized current inhibition by different concentrations of BmK86-P1 on the hKv1.2 channel. Hill equation fitting obtained an IC_50_ value of 28.5 ± 6.3 nM. Each channel was tested more than three times (*n* ≥ 3). The results are indicated as the mean ± S.E.

To investigate the electrophysiological activities of BmK86-P1, the whole-cell patch clamp technique was applied to test the effects of BmK86-P1 on multiple potassium channels. The hKv1.1, hKv1.2, hKv1.3, hKv1.6, hKv3.2, mKv4.1, rKv4.2, rKv4.3 and hERG channels were overexpressed in HEK293T cells, then the inhibitory effects of BmK86-P1 at 1 μM on these potassium channels were measured. The results indicated that 1 μM BmK86-P1 inhibited 4.4 ± 2.6% of hKv1.1 channel currents, 88.6 ± 2.2 of hKv1.2 channel currents, 52.1 ± 3.1% of hKv1.3 channel currents, 11.0 ± 1.2% of hKv1.6 channel currents, 3.8 ± 0.7% of hKv3.2 channel currents, 3.4 ± 1.1% of rKv4.1 channel currents, 11.0 ± 1.8% of rKv4.2 channel currents, 4.9 ± 0.3% of mKv4.3 channel currents and 13.3 ± 3.3 of hERG channel currents (Figure 3A–J). To further investigate the affinity of BmK86-P1 inhibiting the hKv1.2 channel, a dose-dependent study was performed by examining the inhibitory effects of different concentrations of BmK86-P1. The dose–response curve, which was fitted with a Hill equation, showed that BmK86-P1 could inhibit the hKv1.2 channel with an IC_50_ value of 28.5 ± 6.3 nM (Figure 3K,L). These data indicated that the BmK86-P1 peptide was a potent and selective hKv1.2 channel blocker.

### 2.3. Functional Residues in the Channel Pore Region Responsible for BmK86-P1 Binding

**Figure 4:**
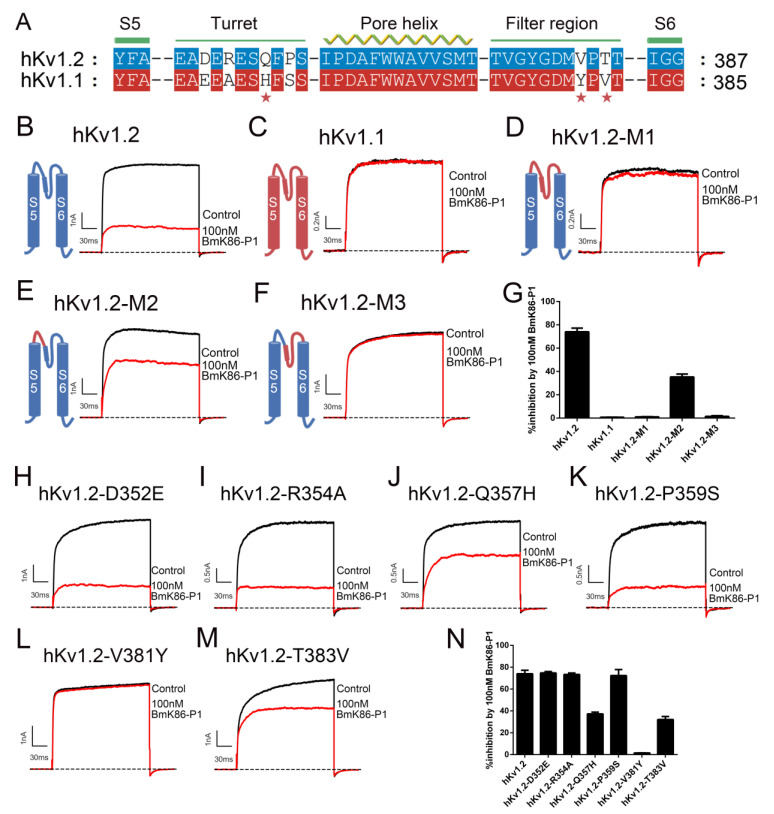
Mapping the site of hKv1.2 channel for BmK86-P1 peptide. (**A**) Sequence alignment of pore regions between hKv1.1 and hKv1.2 channels. (**B**–**F**) Schematic diagrams (left) of potassium channels and representative current traces of the potassium channels in the absence and presence of 100 nM BmK86-P1 (right). (**G**) The inhibitory rates of 100 nM BmK86-P1 on hKv1.2 and hKv1.1 and chimeric channels. BmK86-P1 (100 nM) inhibited 74.1 ± 3.2% of the hKv1.2 channel currents, 0.7 ± 0.1% of the hKv1.1 channel currents, 1.0 ± 0.2% of the hKv1.2-M1 chimeric channel currents, 35.3% ± 2.5% of the hKv1.2-M1 chimeric channel currents and 1.5 ± 0.6% of the hKv1.2-M3 chimeric channel currents. (**H**–**M**) Blocking effects of 100 nM BmK86-P1 on hKv1.2 channel mutants. (**N**) Average inhibition rates by 100 nM BmK86-P1 on hKv1.2 channel mutants. BmK86-P1 (100 nM) inhibited channel currents of 74.8 ± 1.2% for hKv1.2-D352E, 73.3 ± 1.3% for hKv1.2-R354A, 37.3 ± 1.5% for hKv1.2-Q357H, 72.4 ± 5.5% for hKv1.2-P359S, 1.6 ± 0.1% for hKv1.2-V381Y and 32.1 ± 2.8% for hKv1.2-T383V. Each channel was tested at least three times (*n* ≥ 3). The results are shown as the means ± S.E.

The binding interface of potassium channels for scorpion toxins is the pore region, which includes the turret and filter regions [10,11]. We first investigated whether BmK86-P1 peptide binds to the pore region of the hKv1.2 channel. Based on the significantly different binding affinities of BmK86-P1 toward the hKv1.2 and hKv1.1 channels, the chimeric channel hKv1.2-M1 was constructed, in which the S5-S6 linker of the hKv1.2 channel was replaced by the equivalent region of the hKv1.1 channel (Figure 4A–D). Subsequent electrophysiological experiments indicated that BmK86-P1 showed no inhibitory activity on hKv1.2-M1 (Figure 4D). This result revealed that the pore region of the hKv1.2 channel was responsible for the binding of BmK86-P1.

The turret and filter region of potassium channels have different effects on the toxin binding for different scorpion toxins [3,12,13,14]. To investigate the importance of these two regions of potassium channels in interacting with BmK86-P1, another two channel chimeras (i.e., hKv1.2-M2 and hKv1.2-M3) were constructed, in which the turret or the filter region of the hKv1.2 channel was substituted by the equivalent region of the hKv1.1 channel. Subsequent electrophysiological experiments showed that the affinity of BmK86-P1 for these two channel chimeras decreased dramatically, and hKv1.2-M3 exhibited nearly no inhibition by 100 nM BmK86-P1 (Figure 4E–G). These data indicated that BmK86-P1 could interact with the turret and filter region of the hKv1.2 channel, while the filter region of the hKv1.2 channel played a more essential role in BmK86-P1 binding.

To determine the functional sites of the hKv1.2 channel responsible for the selectivity of BmK86-P1, six hKv1.2 mutants were obtained based on sequence differences between the pore regions of the hKv1.2 and hKv1.1 channels (Figure 4A). Then, the activities of 100 nM BmK86-P1 on these six mutant channels were tested. The results indicated that the three hKv1.2 mutants (hKv1.2-Q357H, hKv1.2-V381Y and hKv1.2-T383 V) were differentially blocked by BmK86-P1 compared with the wild-type hKv1.2 channel, and hKv1.2-V381Y exhibited nearly no inhibition by 100 nM BmK86-P1 (Figure 4J,L–N). The other three mutant channels (hKv1.2-D352E, hKv1.2-R354A and hKv1.2-P359S) still kept similar sensitivities toward BmK86-P1 compared with the wild-type hKv1.2 channel (Figure 4H,I,K,N). These data revealed that the Val^381^ in the filter region of the hKv1.2 channel was the critical determinant of peptide selectivity to hKv1.2 over hKv1.1, and two residues (Gln^357^ and Thr^383^) in the pore region of the hKv1.2 channel also affected BmK86-P1 binding.

### 2.4. Functional Sites of BmK86-P1 Identified by Alanine-Scanning Mutagenesis

**Figure 5:**
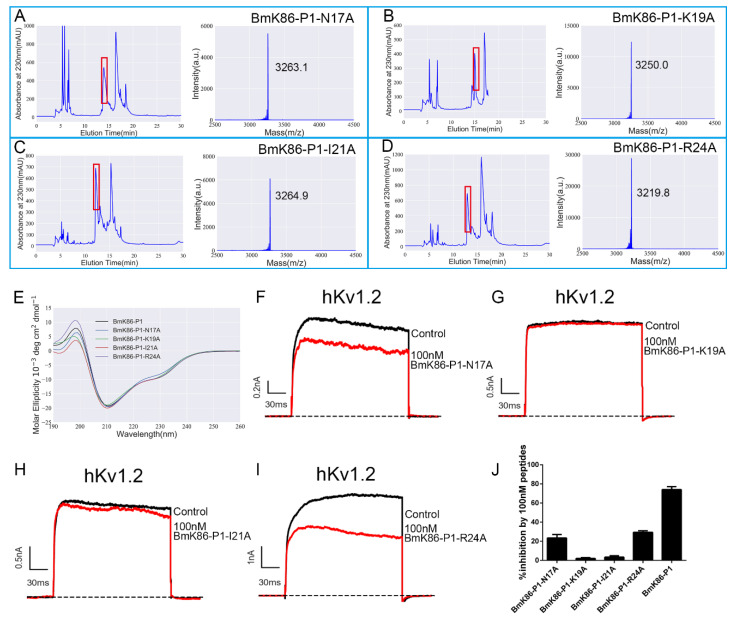
Structure and function relationship of BmK86-P1 peptide on the hKv1.2 channel. (**A**–**D**) Peak profile of four mutants of peptide BmK86-P1 purified by high-performance liquid chromatography (left), in which the part containing mutants of peptide BmK86-P1 are indicated by the red box. Mass spectrometry analysis of these four mutants of peptide BmK86-P1 (right). (**A**) BmK86-P1-N17A; (**B**) BmK86-P1-K19A; (**C**) BmK86-P1-I21A; and (**D**) BmK86-P1-R24A. (**E**) Circular dichroism spectra of peptide BmK86-P1 and its mutants. (**F**–**I**) Blocking effects of BmK86-P1 mutants at 1 μM on hKv1.2 channels current. Results for 100 nM BmK86-P1-N17A (**F**), 100 nM BmK86-P1-K19A (**G**), 100 nM BmK86-P1-I21A (**H**), and 100 nM BmK86-P1-R24A (**I**). (**J**) The inhibitory rates of hKv1.2 channel currents by 100 nM BmK86-P1 and its mutants. Each channel was tested more than three times (n ≥ 3). The results are indicated as the mean ± S.E.

To identify the functional sites of BmK86-P1 involved in the interaction with the hKv1.2 channel, four residues were mutated to alanine respectively. These four residues (i.e., Asn^17^, Lys^19^, Ile^21^ and Arg^24^) were selected based on the orientation of the toxin binding interface induced by the acidic amino acid residue distribution [15,16,17]. These four mutant peptides were successfully obtained by a prokaryotic expression system and then identified by mass spectrometry analysis (Figure 5A–D). Then, we analyzed their secondary structure by CD spectroscopy. The CD spectra of the four mutants (i.e., BmK86-P1-N17A, BmK86-P1-K19A, BmK86-P1-I21A and BmK86-P1-R24A) showed no significant changes compared with wild type BmK86-P1 peptide (Figure 4E), indicating that these four mutant peptides all shared the same overall structural topology as the BmK86-P1 peptide.

The blocking effects of these BmK86-P1 mutant peptides were then evaluated on hKv1.2 channels. BmK86-P1-K19A and BmK86-P1-I21A (100 nM) inhibited only 2.0 ± 0.8% and 3.4 ± 1.5% of the potassium currents mediated by the hKv1.2 channel, respectively (Figure 5G,H). The results showed that the affinities of BmK86-P1-K19A and BmK86-P1-I21A mutants to hKv1.2 channel were dramatic dropped, suggesting that Lys^19^ and Ile^21^ were the key functional residues in the high affinity binding of BmK86-P1 with hKv1.2 channel (Figure 5J). In addition, the two amino acid mutations Asn^17^ and Arg^24^ also caused an obvious decrease in the activities of the mutant peptides on the hKv1.2 channel (Figure 5F,I–J). All these results indicated that these four residues of BmK86-P1 were important in mediating the recognition process toward hKv1.2 channels, and as the classical “functional dyads” of potassium channels inhibiting scorpion toxins, Lys^19^ and Ile^21^ control its affinity for the hKv1.2 channel.

### 2.5. The Absence of the Conserved Cysteine Residue Pattern and Key Residue Deletion in Two New Analogs of BmK86 Results in the Loss of Inhibitory Activities to Potassium Channels

**Figure 6:**
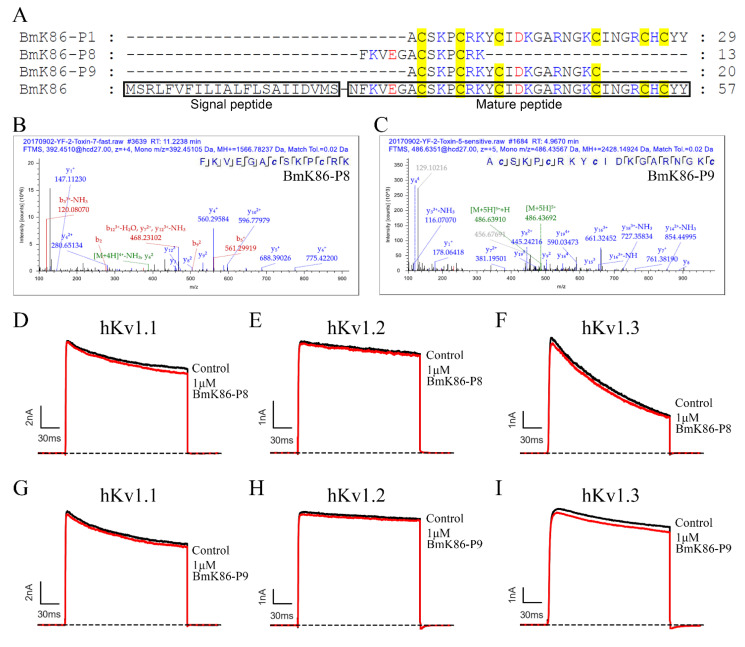
The absence of the conserved cysteine residue pattern and key residue deletion in two new analogs of BmK86 results in the loss of inhibitory activities to the potassium channel. (**A**) Sequence alignment of BmK86 and its three degraded fragments. (**B**–**C**) MS/MS spectra of BmK86-P8 and BmK86-P9. The b-ions and y-ions of the peptides detected in the spectra are shown in red and blue, respectively. The amino acid sequences of peptides are listed, which indicated sequence coverage. (**D**–**F**) Blocking effects of 1 μM BmK86-P8 on hKv1.1, hKv1.2 and hKv1.3 channels. The 1 μM BmK86-P8 could inhibit potassium currents of 0.8 ± 0.3% for hKv1.1 (**D**), 2.5 ± 1.4% for hKv1.2 (**E**), and 3.7 ± 1.6% for hKv1.3 (**F**). (**G**–**I**) Blocking effects of 1 μM BmK86-P9 on hKv1.1, hKv1.2 and hKv1.3 channels. The 1 μM BmK86-P9 could inhibit potassium currents of 3.0 ± 2.1% for hKv1.1 (**G**), 1.8 ± 0.3% for hKv1.2 (**H**), and 3.5 ± 0.3% for hKv1.3 (**I**). Each channel was tested more than three times (*n* ≥ 3). The results are indicated as the means ± S.E.

Another two novel, thermally degraded analogs of BmK86 (i.e., BmK86-P8 and BmK86-P9) were discovered in processed scorpion medicinal material. The b-ions and y-ions of the peptides (i.e., BmK86-P8 and BmK86-P9) detected in the spectra revealed the presence of these two peptides (Figure 6B,C). From the amino acid sequences of these two peptides, we know that the six conserved cysteine residues and the key residues that act on potassium channels were not retained in BmK86-P8 and BmK86-P9 (Figure 6A). To investigate the pharmacological activities of BmK86-P8 and BmK86-P9, these peptides were further prepared by chemical synthesis. According to the pharmacological properties of BmK86 toxin, three potassium channels (i.e., hKv1.1, hKv1.2 and hKv1.3 channels) were selected to detect the inhibitory effects of these two analogs of BmK86. Pharmacological experiments revealed that these two analogs of BmK86 at 1 μM hardly inhibit the current of the hKv1.1, hKv1.2 and hKv1.3 channels (Figure 6D–I). These results indicated that the thermally degraded peptides without six conserved cysteine residues and key functional residues completely lost the inhibitory effects of the wild type toxins on potassium channels.

## 3. Discussion

Thermally processed scorpions have been an important Chinese medicinal material for more than one thousand years. This material is used to treat a variety of diseases [1], which indicates that there are many active substances in processed scorpion medicinal material that can be used to treat diseases. However, until now, we have known very little about the active substances in processed scorpion medicinal material. Recently, this medicinal material was reported to contain a variety of potassium channel-interacting peptides by mass spectrometry-based proteomics, and some of them exhibited high affinity to potassium channels [2,3]. In view of the close relationship between potassium channels and diseases [15,16,17,18], it is essential to characterize the potassium channel-interacting peptides in the processed scorpion medicinal material.

In this study, a novel peptide, BmK86-P1, was found in processed scorpion medicinal material by proteomics. Through sequence comparison, we found that six amnio acids were missing from the N-terminus of the BmK86-P1 peptide compared with the BmK86 toxin, which indicated that BmK86-P1 was derived from the degradation of scorpion toxin BmK86 (Figure 1). The peptide BmK86-P1 contained six conserved cysteine residues, indicating that it could form a 3D structure stabilized by three pairs of disulfide bonds. The pharmacological experiments revealed that BmK86-P1 was a highly potent and selective inhibitor of the hKv1.2 channel with IC_50_ values of 28.5 ± 6.3 nM. Given the crucial role of Kv1.2 in ataxia and epilepsy [4,5,19,20,21], BmK86-P1 might be a potential drug lead for these diseases. Therefore, the interaction mechanism between BmK86-P1 and hKv1.2 channel was further studied. Potassium channel chimeras and mutagenesis experiments indicated that the filter region and the turret of the hKv1.2 channel were all involved in the binding of BmK86-P1, and the channel filter region played a more important role (Figure 4). There were three amino acids in the pore region of the hKv1.2 channel involved in the binding of BmK86-P1 (Figure 4), which was different from the way the Kv1.2 channel interacts with two reported Kv1.2 channel-inhibitory scorpion toxins, mesomartoxin and BmKcug2 [3,22]. These results revealed a new mechanism by which scorpion toxins interact with the hKv1.2 channel. In addition, the extensive mutagenesis revealed that four amino acids (i.e., Asn^17^, Lys^19^, Ile^21^ and Arg^24^) in BmK86-P1 were all involved in the interaction with the hKv1.2 channel, and two critical amino acids (i.e., Lys^19^ and Ile^21^) of BmK86-P1 were found to determine the activity on the hKv1.2 channel (Figure 5).

The BmK86 toxin gene was first discovered from the venom gland cDNA library of *Mesobuthus martensii* Karsch. It encodes a signal peptide with 22 amino acid residues and a mature toxin with 35 residues (Figure 6A) [9]. The stretch of six amino acids in the N-terminus of BmK86 was thought to be a signal peptide rather than the N-terminal portion of the mature peptide [22]. The thermally degraded analogs (i.e., BmK86-P8) of BmK86 in processed scorpion medicinal material revealed that the six amino acids in the N-terminus of BmK86 indeed belonged to the mature peptide. BmK86 could inhibit the current of the Kv1.3 channel with IC_50_ values of 150 ± 57 nM, while BmK86-P1 could inhibit the current of Kv1.3 channel with IC_50_ values of 596.8 ± 114.3 nM [2,9]. These results implied that the N-terminal amino acids of scorpion toxins could affect the pharmacological activities. The insensitivities of potassium channels in response to the BmK86-P8 and BmK86-P9 peptides indicated that conserved cysteine residues and key functional residues were essential for potassium channel-blocking peptides. 

**Figure 7:**
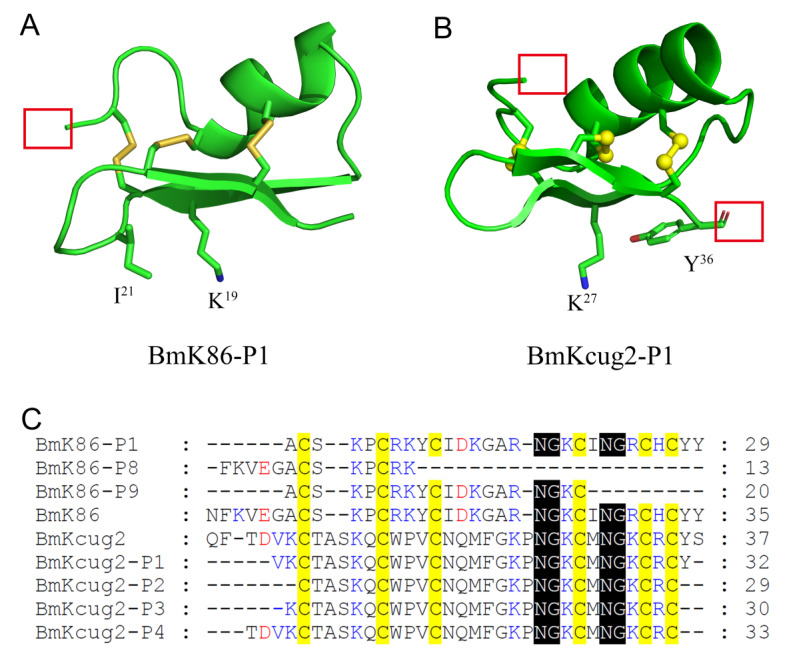
The comparison of seven degraded peptides with or without six conserved cysteine residues from the thermally processed traditional Chinese medicine *Buthus martensii* Karsch. (**A**) The 3D structure of BmK86-P1. The functional amino acids are highlighted. The degraded fraction is indicated by the red box. (**B**) The 3D structure of BmKcug2-P1. The functional amino acids are highlighted. The degraded fraction is indicated by the red box. (**C**) Sequence alignment for BmK86, BmKcug2 and their analogs.

In our previous study, we found that the BmKcug2 toxin analog (i.e., BmKcug2-P1), which undergoes degradation at both the N-terminus and C-terminus of BmKcug2 while maintaining six conserved cysteine residues, and the classical “functional dyad” of potassium channel-inhibiting scorpion toxins, could still act on potassium channels (Figure 7B, C) [3]. However, another three analogs (i.e., BmKcug2-P2, BmKcug2-P3 and BmKcug2-P4 with six conserved cysteine residues) could not effectively inhibit potassium channels due to the loss of one more amino acid residue at the C-terminus of BmKcug2 toxins [3]. Here, the BmK86 toxin analog (i.e., BmK86-P1 with six conserved cysteine residues) potently inhibited hKv1.2 channel currents (Figure 7A,C) when it missed six amino acids in the N-terminus of BmK86. The sequence alignment of BmK86 and BmKcug2 indicated that the peptide C-terminus was more conserved than the N-terminus (Figure 7C). We also found that the peptide binding interfaces were located at the C-terminus of these two peptides (Figure 7A,B). These structural and functional features indicated that the C-terminus of the peptides were more important to their function in interacting with potassium channels. These findings indicated that many novel potassium channel-inhibiting peptides could be acquired by degrading the N-terminus of other scorpion toxins.

In conclusion, this work not only uncovered a novel peptide, BmK86-P1, which is highly potent and selective for the hKv1.2 channel in thermally processed scorpion medicinal material, but also revealed the mechanism by which BmK86-P1 interacts with potassium channels. Moreover, we found that the C-terminus and the conserved six cysteine patterns of scorpion peptides played important roles in interacting with potassium channels. This work could stimulate the discovery of more potassium channel-acting peptides from processed scorpion medicinal material and enriches our understanding of scorpion toxin-potassium channel interactions.

## 4. Materials and Methods

### 4.1. LC-MS/MS Analysis

HCD MS/MS technology was applied to identity potassium channel-inhibiting scorpion toxins in processed scorpion medicinal material [2]. We adopted a two-column system for all analyses. First of all, desalted peptides were resuspended in Solvent A (0.1% formic acid). Then, a trap column (5 μm; 100 μm i.d. × 2 cm Reprosil-Pur C18-AQ) was applied to load on the peptides and an analytical column (3 μm; 75 μm i.d. × 25cm ReprosilPur C18-AQ) was applied to separate on. The HPLC gradient was as follows: a linear gradient of 3% to 8% solvent B (0.1% FA in ACN) over 5 min, 8% to 20% solvent B over 37 min, 20% to 30% solvent B over 6 min, 30% to 90% solvent B over 2 min and 90% solvent B for 15 min at a constant flow rate of 250 nL/min. Full-scans with a resolution of 120,000 were acquired at m/z 200 in the orbitrap for data-dependent acquisition (DDA). In each MS scan, the 15 most abundant ions were selected and fragmented at a resolution of 60,000. The normalized collision energy (NCE) was set to 27.

### 4.2. Construction of Expression Vector

We amplified fragments of BmK86-P1 by the classical overlapping PCR strategy [13]. Then the PCR products were digested by EcoR I and Xho I. The fragments after digestion were then inserted into the pET-32a expression vector. The BmK86-P1 mutants were produced by a QuikChange Site-Directed Mutagenesis Kit (Stratagene, CA, USA) based on the wild type pET-32a-BmK86-P1 plasmid. The recombinant plasmids and its mutants were transformed into competent Escherichia coli Rosetta (DE3) cells for expression after sequence confirmation.

### 4.3. Expression and Purification of Peptides

We expressed recombinant BmK86-P1 and its mutants according to the previous procedure [18]. After the recombinant plasmids of BmK86-P1 and its mutants successfully transformed into *E. coli* Rosetta (DE3) cells, these cells were cultured in the LB medium with ampicillin at 37 °C. Then, 1 mM isopropyl β-D-thiogalactopyranoside (IPTG) was added to induce protein expression at 25 °C. The bacterial cells were harvested and resuspended in a chilled 20 mM imidazole buffer (pH = 7.9). They were cracked by ultrasonic bath and the fusion protein was purified through His-Tag affinity chromatography. The purified fusion proteins were digested by enterokinase (Sangon Biotech, Shanghai, China) at 25 °C for 12 h and then the target peptides were separated through high-performance liquid chromatography. The molecular weight of the peptides was confirmed by matrix-assisted laser desorption ionization time-of-flight mass spectrometry (MALDI-TOF-MS) [23].

### 4.4. Potassium Channels and Cell Culture and Transfection

All potassium channel expression vectors were given by others or constructed by ourselves [3]. HEK293T cells were cultured in Dulbecco’s modified Eagle medium (Gibco, Pittsburgh, PA, USA) with 10% fetal bovine serum and penicillin/streptomycin (100 units/mL) in a 5% CO_2_ incubator at 37 °C. Potassium channel expression vectors and pEGFP-N1 were co-transfected into HEK293T cells using TurboFect in vitro Transfection Reagent (Thermo Scientific, Waltham, MA, USA) according to the manufacturer’s instructions. The potassium currents were recorded after transfection for 1 to 3 days.

### 4.5. Circular Dichroism (CD) Spectroscopy

The secondary structures of BmK86-P1 and its mutants in water were measured by circular dichroism (CD) spectroscopy as previously described using a ChirascanTM V^100^ spectrometer (Applied Photophysics, Surrey, UK) [24]. All the peptides were dissolved into ddH_2_O at a concentration of 0.2 mg/mL. At a range of wavelengths from 180 to 260 nm, the absorption spectra of peptides were recorded at room temperature. The speed scanning was 1 nm/s with a response time of 1 s. The CD spectra of peptides were obtained from the average of three scans after subtracting the blank spectrum for ddH_2_O, and the results were displayed as the mean residue weight (MRW) molar ellipticity [θ] (deg·cm^2^·dmol^−1^).

### 4.6. Electrophysiological Recordings

The channel currents were measured and recorded by whole-cell patch clamp technology according to previously published references [25,26,27]. The internal patch pipette solution and the bath solution were prepared as previously described [3]. All the test peptides were dissolved into bath solution supplemented with 0.01% BSA. An MPS-2 multichannel microperfusion system (INBIO Inc., Wuhan, China) was applied to change the external recording bath solution. The infusion time of the peptides should not exceed 20 min to maintain good cell conditions during electrophysiological recordings. The channel currents were recorded at room temperature (~25 °C) using an EPC 10 patch clamp amplifier. The results were presented as the means ± S.E., and in every sample at least three cells (*n* ≥ 3) were examined. 

### 4.7. Data Analysis

The ClampFit (Molecular Devices, Sunnyvale, CA, USA) and SigmaPlot software (IBM SPSS, Chicago, IL, USA) were used to analyze the recorded data. The dose–effect relationship between the inhibitory effects of the channel currents and the peptides was fitted by the modified Hill equation: I_sample_/I_control_ = 1/{1 + [sample]/IC_50_}, in which I is the peak current, IC_50_ is the half-maximum inhibition concentration, and [sample] represents the concentration of peptides. The parameter to be fitted was the concentration of the half inhibitory concentration (IC_50_).

## Data Availability

All data supporting the results can be found within the manuscript.
